# Heart Failure and Stroke: A Narrative Review

**DOI:** 10.3390/jcm14176044

**Published:** 2025-08-26

**Authors:** Takehiro Katano, Hitoshi Mori, Satoshi Suda

**Affiliations:** 1 Department of Neurology, Nippon Medical School, Tokyo 113-8602, Japan; suda-sa@nms.ac.jp; 2 Department of Cardiology, Saitama Medical University International Medical Center, Saitama 350-1298, Japan; zin_ndmc@yahoo.co.jp

**Keywords:** heart failure, stroke, atrial fibrillation

## Abstract

The global aging population has led to a growing prevalence of heart failure (HF), signaling a new era often referred to as the HF pandemic. HF is strongly associated with both ischemic and hemorrhagic stroke, contributing to the global burden of cerebrovascular disease. In particular, ischemic stroke is frequently observed in patients with HF due to the common coexistence of atrial fibrillation (AF). Given that stroke and HF are both major causes of morbidity and mortality worldwide, a comprehensive understanding of their interrelationship is essential. In 2021, HF was redefined as “a clinical syndrome characterized by symptoms and signs resulting from structural and/or functional cardiac abnormalities, accompanied by current or prior evidence of elevated natriuretic peptides and/or objective findings of pulmonary or systemic congestion,” and it is now classified according to ejection fraction. Among these categories, heart failure with reduced ejection fraction (HFrEF) has been the focus of extensive research, and its treatment has significantly advanced with the development of the so-called “Fantastic Four” pharmacologic therapies. A deeper understanding of the pathophysiological interplay between HF and stroke is crucial to inform future research and improve clinical practice. This review aims to comprehensively summarize the pathophysiological and clinical interrelationship between heart failure and stroke and to provide updated insights for future research and clinical management.

## 1. Introduction

The global prevalence of heart failure (HF) continues to increase with the aging population, resulting in what is now termed a heart failure pandemic. HF significantly contributes to the global burden of stroke and remains a major cause of mortality worldwide. Historically, the condition lacked a universally accepted definition, relying instead on the diagnostic criteria established in the Framingham study [1]. However, in 2021, heart failure was redefined as “a clinical syndrome characterized by symptoms and signs resulting from structural and/or functional cardiac abnormalities, accompanied by current or prior evidence of elevated natriuretic peptides and/or objective findings of pulmonary or systemic congestion” [2]. Currently, heart failure is categorized based on ejection fraction as assessed through echocardiography. These classifications include heart failure with reduced ejection fraction (HFrEF) defined as an ejection fraction below 40%, heart failure with mildly reduced ejection fraction with values between 40% and 50%, and heart failure with preserved ejection fraction (HFpEF) for an ejection fraction of 50% or higher. Recent advances in treatment have significantly improved management strategies for these conditions. This article explores the relationship between heart failure and stroke, focusing particularly on cerebral infarction and atrial fibrillation (AF). The objectives of this review are to provide a comprehensive synthesis of current knowledge on the association between HF and stroke, elucidate their overlapping risk factors and mechanisms, and discuss treatment strategies based on recent evidence. This review is presented as a contemporary narrative synthesis, drawing on recent clinical guidelines, randomized controlled trials, observational studies, and key review articles to provide a comprehensive overview of the relationship between heart failure and stroke.

## 2. Mechanism of Heart Failure

**Figure 1:**
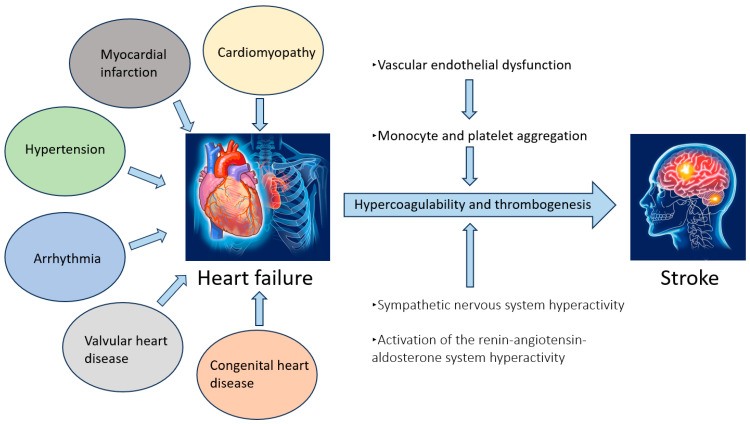
Relationship between heart failure and stroke.

HF is a complex clinical syndrome characterized by the inability of the heart to pump blood effectively to meet the metabolic demands of the body. The pathophysiology of HF involves multiple mechanisms, including systolic dysfunction, diastolic dysfunction, neurohormonal activation, and structural remodeling. Systolic dysfunction leads to an increase in left ventricular end-diastolic volume and pressure, resulting in a reduced ejection fraction. Diastolic dysfunction impairs left ventricular filling. Excessive activation of the sympathetic nervous system and the renin–angiotensin–aldosterone system causes vasoconstriction and sodium and water retention, increasing the workload on the heart. Chronic cardiac stress or injury promotes myocardial hypertrophy and fibrosis, leading to structural and functional changes in the heart and a decline in cardiac function. Background diseases include hypertension, cardiomyopathy, myocardial infarction, valvular disease, arrhythmia, and congenital heart disease (Figure 1).

## 3. Epidemiology of Heart Failure

The global prevalence of HF is estimated to be around 37.7 million cases [3], with this number continuing to rise. Key risk factors for heart failure include aging, hypertension, reduced left ventricular ejection fraction, diabetes, AF, anemia, and chronic kidney disease [4], with aging being a particularly significant global contributor. In the United States, the incidence of new HF increases markedly with age. Among individuals in their 60s, the annual incidence is 1.3% for men and 0.7% for women. This rises to 4.0% and 2.2% among those in their 70s, respectively, and to 8.3% and 7.8% among those in their 80s, respectively [5]. Similarly, Japan, a super-aging society, which is aging earlier than other nations, has seen a substantial increase in HF cases among older individuals. In 1970, the population aged 65 or older with heart failure was approximately 50,000, but, by 2020, this number had surged to an estimated 350,000, representing a seven-fold increase [6].

Gender and racial differences significantly influence the onset and progression of heart failure. According to the European Rotterdam Study, women experience heart failure less frequently than men; however, the incidence tends to surpass that of men in women over 70 years of age [5]. Similarly, the American Framingham Study found that women develop heart failure at older ages and generally have better prognoses than men [7]. The Multi-Ethnic Study of Atherosclerosis highlighted racial disparities in heart failure incidence, reporting an annual rate of 4.6 cases per 1000 individuals among African Americans, compared to 2.4 among Caucasians and just 1.0 among Asians, particularly Chinese Americans [8]. Regarding prognosis, the overall annual mortality rate among heart failure patients is approximately 10%, increasing to 15% for those with HFrEF [9]. Hospitalization further exacerbates mortality risks, while in-hospital mortality rates vary by country, ranging from 2.0% to 7.0%. Additionally, the mortality rate within 30 days post-hospitalization is notably high, ranging from 12% to 26% [10].

## 4. Epidemiology of Heart Failure and Stroke

Heart failure significantly elevates the overall risk of stroke, encompassing all types, including cerebral infarction, intracranial cerebral hemorrhage (ICH), and subarachnoid hemorrhage (SAH). In a nationwide cohort study involving over 289,000 patients with heart failure, the adjusted stroke rate ratios (aSRRs) for ischemic stroke, ICH, and SAH were significantly elevated compared to those in the general population. In the short term (within 30 days), the aSRRs were 5.08 for ischemic stroke, 2.13 for ICH, and 3.52 for SAH. In the long term (over a 30-year follow-up), the risks remained elevated for ischemic stroke (aSRR 1.54) and ICH (aSRR 1.37), whereas the increase in SAH risk was not statistically significant (aSRR 1.13; 95% CI: 0.95–1.35) [11]. Consequently, the increasing prevalence of HF is expected to drive a corresponding rise in stroke cases. The annual incidence of stroke varies with the severity of HF, averaging 1.5% in patients with mild-to-moderate HF [12] and rising to 4% in those with severe HF [13]. From a cardiac function perspective, the annual stroke risk is 1.2% in HFrEF patients with sinus rhythm and 1.0% in HFpEF patients with sinus rhythm. However, the presence of atrial fibrillation significantly increases stroke risk, with annual rates of 1.6% in HFrEF and 1.8% in HFpEF. Although stroke incidence rates are comparable between HFpEF and HFrEF [14], they are consistently higher in patients with AF than in those in sinus rhythm [15,16]. Furthermore, HF patients are approximately four times more likely to develop cerebral infarction than healthy individuals [17], underscoring the likelihood of a substantial increase in cerebral infarction incidence in the future.

## 5. Pathophysiological Link Between Heart Failure and Stroke

As mentioned in the previous section, heart failure increases the risk of all types of stroke, including not only ischemic stroke but also hemorrhagic stroke. Several mechanisms have been proposed to explain this association.

In patients with chronic heart failure, neurohormonal activation—particularly of the sympathetic nervous system and the renin–angiotensin–aldosterone system (RAAS)—plays a pivotal role in disease progression. Reduced cardiac output stimulates the baroreceptor-mediated activation of these systems, leading to increased circulating levels of norepinephrine, angiotensin II, and aldosterone. This neurohormonal upregulation promotes systemic vasoconstriction, sodium retention, and, notably, the production of inflammatory cytokines, such as interleukin-6 (IL 6) and tumor necrosis factor-alpha (TNF-α), thereby sustaining a state of chronic low-grade inflammation [18].

Chronic systemic inflammation is increasingly recognized as a critical contributor to cerebral small-vessel disease (SVD). According to Wardlaw et al., pro-inflammatory cytokines such as IL-1β, IL-6, and TNF-α can impair endothelial cell function, reduce nitric oxide availability, and disrupt the blood–brain barrier (BBB), leading to the leakage of plasma proteins and immune cells into the brain parenchyma. These processes promote SVD. Persistent endothelial dysfunction and oxidative stress may also impair the neurovascular unit, further exacerbating tissue damage and increasing ischemic vulnerability [19].

Neurovascular coupling (NVC) denotes the moment-to-moment matching of regional cerebral blood flow to local neuronal activity and is mechanistically distinct from global cerebral autoregulation or CO_2_-driven cerebrovascular reactivity. In chronic HF, systemic inflammation, endothelial dysfunction, microvascular rarefaction, and neurohormonal activation may blunt NVC, limiting the brain’s ability to augment flow during task-evoked demand. Clinically, impaired NVC has been associated with cognitive decline, silent cerebral infarcts, and worse post-stroke outcomes. Using visually evoked transcranial Doppler protocols provided direct evidence of blunted posterior cerebral artery flow responses to visual stimulation in HF patients compared with controls, supporting the concept that NVC impairment represents a critical pathophysiologic link between HF and brain vulnerability [20].

Moreover, vascular fragility is caused by fluctuations in blood pressure. Patients with heart failure often experience repeated episodes of hypotension and hypertension, which can lead to blood pressure variability (BPV). BPV has emerged as an important factor contributing to vascular fragility, particularly in the cerebral circulation. Repeated fluctuations in blood pressure impose mechanical stress on vessel walls, leading to endothelial dysfunction, impaired vascular repair, and structural damage to small arteries. This is especially relevant in the brain, where small penetrating vessels are vulnerable to hemodynamic stress. Increased BPV has been associated with the development of cerebral small-vessel disease, including microbleeds and white matter lesions, which are known precursors to ICH [21]. In addition, the frequent coexistence of atrial fibrillation in these patients necessitates the use of anticoagulant therapy for stroke prevention, which, in turn, increases the risk of bleeding. Other contributing factors include the presence of degenerative vascular conditions, such as cerebral amyloid angiopathy.

One mechanism by which heart failure increases the risk of cerebral infarction is the heightened activation of Virchow’s triad, which includes blood flow stagnation, vascular endothelial damage, and a hypercoagulable state.

Decreased wall motion and enlargement of the left atrium and ventricle in HF patients contribute to blood flow stagnation. Additionally, arteriosclerosis, a common cause of HF, damages vascular endothelial cells and reduces the production of nitric oxide by the endothelium, facilitating monocyte and platelet aggregation within the vascular wall. Chronic heart failure also triggers sympathetic nerve activation and the renin–angiotensin–aldosterone system, promoting a hypercoagulable state. These factors collectively increase the risk of thrombus formation and embolism.

In HFrEF, a low left ventricular ejection fraction and wall motion abnormalities can lead to regional blood stasis and even mural thrombus formation, especially after large myocardial infarctions [22]. Left ventricular thrombus was identified in approximately 9% of patients with heart failure and an LVEF of ≤35% [23].

Cardiogenic cerebral embolism is more common in HFrEF because ventricular dilatation and reduced ventricular contractility increase the likelihood of thrombus formation and embolization. Indeed, HF due to ischemic or valvular causes has been linked to higher rates of cardioembolic strokes [14]. However, HFpEF patients often have a high burden of atherosclerotic risk factors, such as advanced age, hypertension, and metabolic comorbidities. These factors may predispose them to stroke subtypes such as small-vessel occlusion (lacunar strokes) and large-artery atherosclerosis [14].

**Figure 2:**
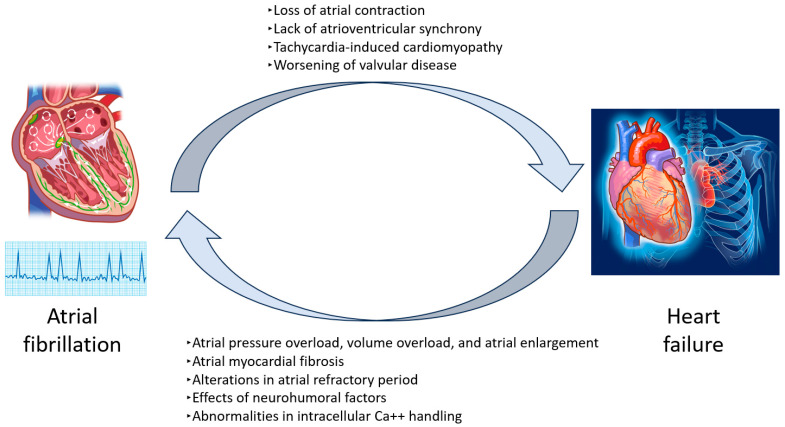
Relationship between atrial fibrillation and heart failure.

Among the important factors contributing to ischemic stroke in patients with HF is the coexistence of AF. The frequent occurrence of AF in HF further amplifies the risk of cerebral infarction. HF and AF share numerous risk factors, including aging, hypertension, diabetes, and chronic kidney disease. Each condition also predisposes patients to the other, underscoring their close interrelation [4,24]. HF patients are approximately five times more likely to develop AF [25], while about 30% of individuals with AF eventually develop heart failure [26]. Compared to healthy individuals, the risk of stroke is 3.7 times higher in those with heart failure, 4.2 times higher in those with AF, and 5.3 times higher in individuals with both conditions [17] (Figure 2).

## 6. Risk Factors and Predictive Models for Stroke

As previously mentioned, one of the major contributors to the risk of stroke in patients with HF is the coexistence of AF. Therefore, screening for AF and assessing embolic risk are of critical importance. AF has diverse etiologies, and its characteristics vary depending on age [27], sex [28], ethnicity [29], and genetics [30,31], suggesting regional differences and the need for region-specific evaluation strategies. For example, in Japan, instead of conventional scores such as CHADS_2_ [32] and CHA_2_DS_2_-VASc [33], the HELT-E_2_S_2_ score has been proposed for assessing the risk of embolism [34], and, rather than the HAVOC score [35] and Brown ESUS-Af score [36], the LOOK-AF score has been developed to predict the presence of AF in patients with cryptogenic stroke [37], both of which are being applied in clinical practice.

The risk of ischemic stroke in patients with HF has been extensively studied. The CHA2DS2-VASc score has been reported to be a predictor of stroke not only in AF [32] but also in HF. A CHA2DS2-VASc score of 4 or higher has been identified as a predictor of cerebral infarction for HF without AF [38]. In a randomized controlled trial comparing warfarin and aspirin, a risk scoring system for HF patients in sinus rhythm (with an ejection fraction of 35% or less) was developed. This system included factors such as age, oxygen saturation, ejection fraction, hemoglobin level, sex, diastolic blood pressure, diabetes, and a history of stroke, although only an ejection fraction of 15% or less was significantly associated with stroke risk [39,40]. Additionally, another study focusing on patients with sinus rhythm HF (ejection fraction ≤ 35%) and a history of myocardial infarction proposed a scoring system based on age (60–75 years and ≥75 years), Killip class (3 or 4), estimated glomerular filtration rate (eGFR ≤ 45 mL/min/1.73 m^2^), hypertension, and history of stroke, which was found to be predictive of stroke risk [41]. Across multiple studies, common risk factors cited include advanced age, reduced cardiac function, and a history of stroke.

## 7. Clinical Outcomes and Prognosis After Ischemic Stroke

Acute ischemic stroke can itself precipitate new or worsening heart failure—a phenomenon referred to as “stroke–heart syndrome.” In large cohort studies, approximately 6.4% of patients developed new-onset HF within 4 weeks of stroke onset, while other cardiovascular complications such as atrial fibrillation (8.8%) and acute coronary syndromes (11.1%) were also frequently observed [42]. These events were independently associated with increased long-term mortality, rehospitalization, and major adverse cardiovascular events [43]. The underlying mechanisms include autonomic dysfunction, systemic inflammation, and stress-induced cardiomyopathy, including Takotsubo syndrome. This bidirectional interaction between the brain and heart adds complexity to the clinical course and prognosis of stroke patients [43].

Ischemic stroke in the context of HF is associated with poorer clinical outcomes and long-term prognosis than stroke in patients without HF. Multiple cohort studies have shown that HF more than doubles short-term mortality after stroke in some analyses, and it increases the risk of stroke complications [11,44]. For instance, a nationwide study in Taiwan reported that prior HF was linked with a ~40% higher odds of death after an ischemic stroke [45]. Similarly, post-stroke complications like pneumonia and sepsis were about 30% more frequent in patients with HF [43], reflecting the frailty and impaired reserve in this population. Other registries have observed an even greater excess mortality, with HF patients experiencing between 2.2 to 4.5 times higher post-stroke mortality than those without HF in certain cohorts [46]. HF is also an independent predictor of worse functional outcomes and disability after stroke [47]. When comparing HFpEF vs. HFrEF in post-stroke prognosis, direct comparative data are limited, but both groups appear vulnerable to adverse outcomes. In a combined analysis of clinical trials, patients with a history of stroke had substantially higher rates of all-cause death and cardiovascular (CV) death in both HFrEF and HFpEF groups than those without a history of stroke [47].

The excess risk was present irrespective of EF category [48], suggesting that, once a stroke has occurred, preserved EF offers little survival advantage. HFpEF patients are typically older with more comorbidities, which might predispose them to non-cardiac deaths after stroke (e.g., due to infections or recurrent strokes), whereas HFrEF patients may have more cardiac deaths. But, overall, any HF combined with stroke portends a worse long-term prognosis than stroke alone. One analysis noted that, among HF patients with prior stroke, subsequent CV events (stroke, HF hospitalizations, and MI) were 33–50% more frequent than among those without prior stroke [31].

## 8. Management and Treatment Strategies

Recent clinical guidelines, including the 2022 AHA/ACC/HFSA Guideline for the Management of Heart Failure, the 2024 AHA/ASA Guideline for the Primary Prevention of Stroke, and the 2024 Japanese Stroke Society Guidelines, emphasize the importance of integrated management for patients with heart failure and concomitant stroke risk. These documents offer updated recommendations for pharmacological treatment, anticoagulation strategies, and risk stratification in this complex patient population. Incorporating these guideline-based approaches into clinical practice is essential to optimize outcomes and reduce morbidity and mortality associated with both heart failure and stroke [49,50,51]. Cardiogenic embolism is associated with a poorer prognosis than other types of cerebral infarction, and treatment and prevention of cardiogenic cerebral embolism are considered important [52,53]. But, in patients with HF, addressing the HF itself takes precedence over stroke prevention. When AF occurs in conjunction with HF, the risk of mortality increases by two to three times [54], and HF remains the primary cause of death in individuals with AF [13]. Consequently, even when AF coexists with heart failure, treating HF remains the top priority.

### 8.1. Pharmacological Therapies for Heart Failure

Until the 1970s, diuretics were the primary treatment for HFrEF. However, the therapeutic landscape evolved significantly with the introduction of angiotensin-converting enzyme (ACE) inhibitors in the 1980s [55], β-blockers and mineralocorticoid receptor antagonists (MRAs) in the 1990s [56,57,58], and angiotensin receptor–neprilysin inhibitors (ARNIs) and sodium–glucose cotransporter 2 (SGLT2) inhibitors in the 2010s [59,60]. This progression led to the establishment of the “Fantastic Four” treatment strategy. This approach prioritizes the early initiation of β-blockers and SGLT2 inhibitors, followed by the introduction of ARNIs and MRAs, which has been shown to improve patient outcomes [61,62,63,64]. Additionally, newer therapies for heart failure include ivabradine and vericiguat. Ivabradine, unlike β-blockers, does not reduce cardiac contractility or lower blood pressure but instead selectively lowers the heart rate. It is particularly effective in HF patients with a resting heart rate of 75 beats per minute or higher, even when β-blockers are being used. Vericiguat has demonstrated efficacy as an add-on therapy for HFrEF patients experiencing worsening HF when combined with standard HF treatments. These therapies can lead to improvement in cardiac function. Heart failure with improved ejection fraction (HFimpEF) is a recently recognized clinical entity defined as HF in patients with a previously reduced LVEF, typically ≤40%, that subsequently improves to >40% in response to therapy. HFimpEF patients are considered to have a distinct phenotype with a more favorable prognosis than those with persistent HFrEF. And clinical trials and guideline recommendations emphasize that these patients should continue medical therapy, as the withdrawal of treatment may lead to the deterioration of cardiac function and the recurrence of heart failure symptoms [47,48,49]. There is substantial evidence supporting the treatment of HFrEF.

The efficacy of SGLT2 inhibitors has also been demonstrated in HFpEF. Two large, contemporary RCTs—EMPEROR-Preserved (empagliflozin) and DELIVER (dapagliflozin)—demonstrated significant reductions in the composite of cardiovascular death or worsening heart failure across the full EF spectrum, including HFpEF [63,64]. In EMPEROR-Preserved, empagliflozin reduced the primary outcome versus placebo (HR 0.79, 95% CI 0.69–0.90), driven mainly by fewer HF hospitalizations; the effects were consistent with and without diabetes [63]. DELIVER confirmed these findings with dapagliflozin (HR 0.82, 95% CI 0.73–0.92), with benefits again largely from fewer HF events [64]. Reflecting these data, the 2022 AHA/ACC/HFSA Guideline assigns SGLT2 inhibitors a Class 2a recommendation for HFpEF [48], and the 2023 ESC update elevates SGLT2 inhibitors to Class I (LOE A) across HF with mildly reduced or preserved EF [65]. SGLT2 inhibitors significantly reduce the risk of hospitalization for heart failure in this population [63,64]. Other pharmacological therapies for HFpEF, including spironolactone, MRAs, ARNIs, β-blockers, and ACE inhibitors, improved patient-reported symptoms and quality of life in patients with an LVEF closer to the mid-range and have demonstrated more limited or inconsistent benefits [66,67]. Furthermore, SGLT2 inhibitors have been reported to significantly reduce the risk of new-onset atrial fibrillation [68,69]. In terms of stroke, no significant change was observed in the risk of total stroke (including fatal and non-fatal events) with SGLT2 inhibitor use (RR = 0.95; 95% CI, 0.79–1.13; *p* = 0.585). While no efficacy was observed for ischemic stroke, some studies have suggested a potential 50% reduction in the risk of hemorrhagic stroke (RR = 0.49; 95% CI, 0.30–0.82; *p* = 0.007) [70], indicating a promising avenue for future investigation.

Regarding the use of antithrombotic drugs in patients with chronic HF, a meta-analysis comparing antiplatelets and oral anticoagulants in HFrEF patients found that oral anticoagulants reduced the risk of cardiovascular events, including stroke, compared to antiplatelets in patients without AF. However, this benefit came at the cost of a risk of bleeding [71]. As a result, although warfarin may provide benefits for HFrEF patients in sinus rhythm, its routine use is not currently recommended because of the increased bleeding risk. Table 1 shows randomized clinical trials investigating anticoagulant therapy for stroke associated with HF in sinus rhythm. Not all trials demonstrated the effectiveness of anticoagulant therapy [72,73,74,75,76]. Notably, a post hoc analysis of the COMMANDER-HF trial demonstrated that low-dose rivaroxaban (2.5 mg twice daily) significantly reduced the incidence of ischemic stroke or TIA by approximately 32% (adjusted HR 0.68, 95% CI 0.49–0.94), with comparable rates of major bleeding between treatment arms [77].

### 8.2. Pharmacological Therapies for Atrial Fibrillation

**Figure 3:**
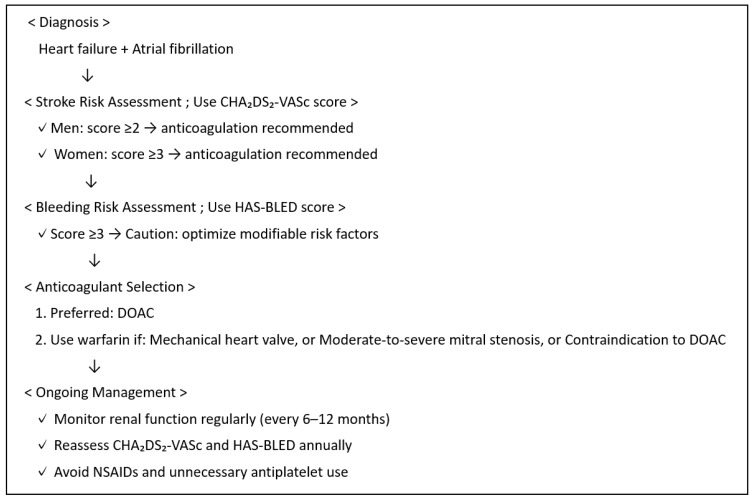
Algorithm for anticoagulation in patients with heart failure and atrial fibrillation. Proposed algorithm for oral anticoagulation in patients with both heart failure and atrial fibrillation (HF + AF). Stroke risk should be assessed using the CHA_2_DS_2_-VASc score, and bleeding risk should be assessed using HAS-BLED. Direct oral anticoagulants (DOACs) are generally preferred over warfarin unless contraindicated. Ongoing monitoring and periodic reassessment are essential to optimize benefit and minimize harm.

Randomized controlled trials such as RELY, ARISTOTLE, ROCKET AF, and ENGAGE AF demonstrated that direct oral anticoagulants (DOACs) are non-inferior to warfarin in preventing stroke or systemic embolism in patients with atrial fibrillation (AF), including those with concurrent heart failure (HF). These studies also showed favorable safety profiles for DOACs compared to warfarin [78,79,80,81] (Table 2). A meta-analysis of these four studies revealed that the DOAC group had a 14% reduction in the incidence of stroke or systemic embolism and a 24% reduction in bleeding events in patients with both AF and HF, compared to the warfarin group, demonstrating both efficacy and safety [82]. Consequently, DOACs are considered a viable option for anticoagulant therapy in patients with HF and AF. However, the efficacy of DOACs in patients with HF in sinus rhythm remains unclear. In a study involving patients with ischemic heart disease and HFrEF (LVEF < 40%), there was no significant difference in composite events (myocardial infarction, stroke, and all-cause mortality) or bleeding complications between the DOAC and placebo groups [76]. Figure 3 shows an algorithm for anticoagulation in patients with HF and AF according to the 2022 AHA/ACC/HFSA Guideline for the Management of Heart Failure and the 2024 AHA/ASA Guideline [49,50]. Importantly, other clinical conditions for which warfarin is recommended include antiphospholipid antibody syndrome and the presence of left ventricular thrombi. In addition, some newer guidelines, such as those from the ESC, seem to prefer the CHA_2_DS_2_-VA score (which excludes the sex category) over the traditional CHA_2_DS_2_-VASc score [83]. The HAS-BLED score is a clinical tool used to estimate the risk of major bleeding in patients with atrial fibrillation who are being considered for anticoagulation therapy. It incorporates the following factors: hypertension, abnormal renal and liver function, stroke, bleeding history or predisposition, labile INR, elderly (age > 65), and the use of drugs or alcohol (each scoring one point). A total score of 3 or more indicates a high risk of bleeding and calls for caution and regular review, but it should not be used alone to exclude patients from receiving anticoagulation therapy [84].

### 8.3. Non-Pharmacological Therapies for Heart Failure

Non-pharmacological therapies have also advanced significantly, including catheter-based and device-based treatments. Catheter-based interventions, such as coronary angioplasty, are employed for ischemic heart disease, a major contributor to HF. Ablation therapies are utilized to manage AF, while transcatheter aortic valve implantation treats structural heart diseases, including valvular and congenital heart conditions. Additionally, since sudden death is a leading cause of mortality in patients with mild-to-moderate heart failure, implantable cardioverter defibrillators have been shown to improve survival outcomes. For patients with refractory HF, left ventricular assist devices may be implanted, either as a bridge to heart transplantation or as destination therapy for those ineligible for transplants. Furthermore, in patients with atrial fibrillation (AF), stroke prevention can also be achieved through left atrial appendage occlusion, performed either via a percutaneous or surgical approach (percutaneous and surgical left atrial appendage occlusion [LAAO]) [85,86]. The primary indication for percutaneous LAAO is stroke prevention in patients with non-valvular AF who have a high thromboembolic risk but are unsuitable for long-term oral anticoagulation due to prior major hemorrhage or other prohibitive bleeding risks. The PROTECT-AF trial demonstrated that the Watchman device was non-inferior to warfarin for the composite endpoint of stroke, systemic embolism, or cardiovascular death, with long-term follow-up suggesting potential superiority in reducing this composite and all-cause mortality, albeit with higher early procedural complication rates [85]. The subsequent PREVAIL trial confirmed non-inferiority for efficacy endpoints and showed improved procedural safety compared to PROTECT-AF [87]. More recently, the PRAGUE-17 trial compared LAAO with direct oral anticoagulants (DOACs) in high-bleeding-risk AF patients and found LAAO to be non-inferior for a composite of stroke, systemic embolism, cardiovascular death, major bleeding, or device/procedure-related complications, with a trend toward lower major bleeding during long-term follow-up [88]. Collectively, these trials support LAAO as an effective alternative to oral anticoagulation in appropriately selected patients, and contemporary guidelines assign it a Class 2a recommendation for patients with a clear contraindication to long-term OAC and a Class 2b recommendation for those with a high bleeding risk despite OAC, emphasizing shared decision-making and individualized patient selection.

These interventions are considered alternatives to prolonged anticoagulation in patients with non-valvular atrial fibrillation who have an indication for long-term anticoagulant therapy. In addition, percutaneous patent foramen ovale (PFO) closure has also been reported to be effective as a preventive therapy for recurrent stroke in patients with cryptogenic stroke [89,90,91]. As many of these are relatively recent interventions with limited long-term evidence, their use should be carefully considered on a case-by-case basis.

### 8.4. Stroke Management in Patients with Heart Failure

Stroke in patients with HF requires specific consideration due to the elevated risks of both thrombotic and hemorrhagic events. When ischemic stroke occurs, intravenous thrombolysis (IV-tPA) remains the standard treatment, but clinicians must assess the bleeding risk carefully, especially in patients with severe HF, advanced age, or AF or those on chronic anticoagulation. It has been reported that, in patients with AF, the use of IV-tPA is associated with a significantly higher risk of symptomatic ICH than in those without AF (OR 1.96, 95% CI 1.23–3.11) [92]. The efficacy and safety of endovascular thrombectomy are not significantly altered by the presence of HF, though outcomes may be worse due to overall frailty. Secondary stroke prevention in HF patients includes the optimization of HF management, blood pressure control, and antithrombotic therapy tailored to the rhythm status. In patients with AF, oral anticoagulation is mandatory. In those with sinus rhythm, anticoagulation remains controversial and should be individualized. Non-pharmacologic strategies, including cardiac rehabilitation and lifestyle modification, should also be implemented to reduce recurrent stroke risk.

## 9. Future Directions and Research Gaps

In recent years, both pharmacological and non-pharmacological treatments for cardiovascular disease have seen significant advancements. Many medical therapies such as ARNIs and SGLT2 inhibitors not only alleviate symptoms but also improve cardiac remodeling and survival. However, achieving a complete cure by addressing the underlying causes remains an unresolved challenge. Cardiovascular disease results from a complex interplay of environmental and genetic factors, and its pathology is not yet fully understood. Nevertheless, recent developments in genomics, as well as in computing and artificial intelligence, have enabled the analysis of these vast and intricate data. Genome analysis is being applied to investigate treatments for single-gene disorders, such as cardiomyopathy and long QT syndrome, which can contribute to HF. Studies have shown that analyzing causative genes in patients with dilated cardiomyopathy may help in selecting appropriate treatments [93]. Furthermore, genomic analysis can assess the risk of multifactorial diseases, such as myocardial infarction and AF, enabling the prediction of prognosis and facilitating disease prevention. Currently, preventive measures for HF are highly effective, making it crucial to focus on understanding its mechanisms, preventing its onset, and reducing the risk of associated strokes. Future advancements in understanding the pathogenesis of HF are expected to lead to molecular-targeted therapies and precision medicine tailored to individual patients.

## 10. Conclusions

HF significantly elevates the risk of stroke, encompassing both ischemic and hemorrhagic types. This increased risk is influenced by factors such as the severity of HF, the presence of atrial fibrillation, and underlying comorbidities. Notably, HF not only heightens the incidence of stroke but also adversely affects short-term mortality and functional outcomes post-stroke. The intricate interplay between HF and stroke underscores the necessity for comprehensive management strategies that address both conditions concurrently. Future research should focus on elucidating the pathophysiological mechanisms linking HF and stroke, refining risk stratification models, and developing targeted interventions to mitigate the compounded risks associated with these prevalent cardiovascular disorders. Therefore, a comprehensive understanding and co-management of both conditions are vital. Stroke risk must be considered proactively in patients with HF, and stroke treatment should be personalized considering the presence and severity of HF. This interplay calls for collaborative cardiology–neurology care.

## Figures and Tables

**Table 1:** Clinical trials investigating anticoagulant therapy with heart failure in sinus rhythm.

Trial Name	LVEF	Study Design	Intervention	Primary Outcome	Result
WASH	≤35%	Open-label, randomized, control trial.	Warfarin vs. aspirin vs. no anti-platelet therapy	Composite outcome of death, non-fatal myocardial infarction, and non-fatal stroke	No significant difference in the primary clinical outcome among the 3 groups
HELAS	≤35%	Double-blind, randomized, placebo-controlled trial.	Warfarin vs. aspirin vs. placebo	Composite of non-fatal stroke, peripheral or pulmonary embolism, myocardial infarction, rehospitalization, worsening heart failure, and all-cause mortality	No significant difference in the primary clinical outcome among the 3 groups
WATCH	≤35%	Double-blind, randomized trial. Double-dummy controlled for anti-platelet therapy or open-label warfarin.	Warfarin vs. aspirin vs. clopidogrel	Composite of all-cause mortality, non-fatal myocardial infarction, and non-fatal stroke	No significant difference in the primary clinical outcome among the 3 groups
WARCEF	≤35%	Double-blind, randomized, double-dummy controlled trial.	Warfarin vs. aspirin	Time to first event in a composite endpoint of ischemic stroke, intracerebral hemorrhage or all-cause mortality	No difference between the 2 groups
COMMANDER-HF	≤40%	Double-blind, randomized, placebo-controlled trial.	Rivaroxaban vs. placebo	Composite of all-cause mortality, non-fatal myocardial infarction, and non-fatal stroke	No difference between the 2 groups

**Table 2:** Summary of major trials on DOACs in patients with HF and AF.

Trial Name	DOAC	Patient Population	Efficacy (Stroke/Systemic Embolism) (vs. Warfarin)	Major Bleeding Risk (vs. Warfarin)	Key Notes
RE-LY	Dabigatran	Non-valvular AF, included HF	Superior (150 mg twice daily)	Similar overall; increased gastrointestinal bleeding at 150 mg dose	Two-dose comparison (110 mg and 150 mg)
ROCKET AF	Rivaroxaban	Non-valvular AF (CHADS_2_ score ≥ 2), included HF	Non-inferior	Similar overall; increased gastrointestinal bleeding	Once-daily dosing High-risk population
ARISTOTLE	Apixaban	Non-valvular AF, included HF	Superior	Lower major bleeding, including lower gastrointestinal bleeding	Once-daily dosing
ENGAGE AF	Edoxiaban	Non-valvular AF, included HF	Non-inferior	Lower major bleeding, especially lower gastrointestinal bleeding	Two doses tested (60 mg and 30 mg)

## Data Availability

The original contributions presented in this study are included in the article. Further inquiries can be directed to the corresponding author.
